# Public beliefs about causes and risk factors for mental disorders: a comparison of Japan and Australia

**DOI:** 10.1186/1471-244X-5-33

**Published:** 2005-09-21

**Authors:** Yoshibumi Nakane, Anthony F Jorm, Kumiko Yoshioka, Helen Christensen, Hideyuki Nakane, Kathleen M Griffiths

**Affiliations:** 1 Department of Social Work, The Faculty of Human Sociology, Nagasaki International University, 2825-7 Huis Ten Bosch-cho, Sasebo-shi, Nagasaki, 859-3298, Japan; 2 ORYGEN Research Centre, Department of Psychiatry, University of Melbourne, Locked Bag 10, Parkville, Victoria 3052, Australia; 3 Centre for Mental Health Research, Australian National University, Canberra, ACT 0200, Australia; 4 Division of Neuropsychiatry, Department of Translational Medical Sciences, Nagasaki University Graduate School of Biomedical Sciences, 1-7-1 Sakamoto, Nagasaki, 852-8501, Japan

## Abstract

**Background:**

Surveys of the public in a range of Western countries have shown a predominant belief in social stressors as causes of mental disorders. However, there has been little direct cross-cultural comparison. Here we report a comparison of public beliefs about the causes of mental disorders in Japan and Australia.

**Methods:**

Surveys of the public were carried out in each country using as similar a methodology as feasible. In both countries, household interviews were carried out concerning beliefs about causes and risk factors in relation to one of four case vignettes, describing either depression, depression with suicidal thoughts, early schizophrenia or chronic schizophrenia. In Japan, the survey involved 2000 adults aged between 20 and 69 from 25 regional sites spread across the country. In Australia, the survey involved a national sample of 3998 adults aged 18 years or over.

**Results:**

In both countries, both social and personal vulnerability causes were commonly endorsed across all vignettes. The major differences in causal beliefs were that Australians were more likely to believe in infection, allergy and genetics, while Japanese were more likely to endorse "nervous person" and "weakness of character". For risk factors, Australians tended to believe that women, the young and the poor were more at risk of depression, but these were not seen as higher risk groups by Japanese.

**Conclusion:**

In both Japan and Australia, the public has a predominant belief in social causes and risk factors, with personal vulnerability factors also seen as important. However, there are also some major differences between the countries. The belief in weakness of character as a cause, which was stronger in Japan, is of particular concern because it may reduce the likelihood of seeking professional help and support from others.

## Background

Mental health researchers view mental disorders as having complex causes involving an interplay of biological, psychological and social factors. However, the public's beliefs about causes are generally less sophisticated. Surveys of the public in a range of Western countries have shown a predominant belief in social stressors as causes of mental disorders. Studies from Australia, Ireland, Germany, Switzerland, UK and USA have found that social factors were most often seen as the causes of depression [1-6], whereas genetic factors were much less frequently endorsed [1-4,6]. Social factors are also seen by the public of Western countries as an important cause of schizophrenia [3,6-8]. While genetic factors are more often seen as a cause for schizophrenia than depression, they are still endorsed much less frequently than social factors [3,6]. Social factors covered in these surveys included stressful life events, traumatic experiences, family problems, and social disadvantage.

Of greater concern is the stigmatizing belief that mental disorders are caused by personal weakness or a character flaw. While this is not a predominant belief, it is fairly common. In the USA, around one-third saw "own bad character" as a cause for both schizophrenia and depression [6], implying a moral judgment of mental disorders. In Australia, around half the population believed "weakness of character" is a cause of both depression and schizophrenia [1], while in Turkey over 60% believed that this is a cause of schizophrenia [8].

While there has been considerable research on public beliefs in Western countries, there has been little research in other parts of the world and little cross-cultural comparison. The beliefs that predominate in Western countries cannot be assumed to apply elsewhere. A comparison of teachers' beliefs about schizophrenia in Japan and Taiwan found that "stress from personal relations" was commonly seen as a cause in both countries, which is similar to the belief in social factors in Western countries [9]. However, the Taiwanese were more likely than the Japanese to believe in "weakness of character", "heredity" and "stress from a disaster" as a cause. A comparison of mainly young adults from Hong Kong and England found that the Hong Kong Chinese were more likely to believe in social factors as the cause of schizophrenia, while the English were more likely to endorse genetic factors [10]. This difference was attributed to the more collectivist nature of Chinese culture. There has also been a comparison of public beliefs in Germany, Russia and Mongolia [11]. In all three countries, psychosocial factors such as stressful life events were predominantly seen as the cause of both depression and schizophrenia, whereas biological causes such as heredity and brain disease were less frequently endorsed. Taken together, these cross-cultural comparisons indicate that a belief in social causes is common in East Asian countries as well as in Western countries.

Here we report a further cross-cultural comparison involving Japan and Australia. This comparison involved surveys in both countries using the same questions about causes and risk factors for four case vignettes: depression, depression with suicidal thoughts, early schizophrenia, and chronic schizophrenia. On the basis of previous research, it might be expected that a belief in social causes would predominate in both countries. However, there are a number of cultural and health system differences between the two countries that might influence responses. Japan places a greater emphasis on hospital care compared to the emphasis on community care in Australia. The Japanese mental health care system has been described as based on the values of minimizing state financial involvement, retaining family responsibility for family members, and social control of individuals who might contribute to social disorder [12]. By contrast, Western systems are more influenced by the values of individual rights, social reintegration and government responsibility [12]. While these differences exist, it is difficult to predict what effect they might have on causal beliefs, so the study was essentially exploratory.

## Methods

### Survey interview

Interview questionnaires comprised a common core of questions that would allow comparisons between countries, and a country-specific component to allow investigation of issues particular to each country [13]. Copies of the Japanese questionnaire are available from YN and of the Australian questionnaire from AFJ. The interview was based on a vignette of a person with a mental disorder. On a random basis, respondents were shown one of four vignettes: a person with major depression, one with major depression together with suicidal thoughts, a person with early schizophrenia, and one with chronic schizophrenia. All vignettes were written to satisfy the diagnostic criteria for either major depression or schizophrenia according to DSM-IV and ICD-10. The vignette with depression and the one with early schizophrenia were written to satisfy at a minimal level these diagnostic criteria, so that we could ascertain the public's reaction to cases of developing disorder that had reached the point where intervention was needed. The vignette of the person with depression together with suicidal thoughts was identical to the depression vignette in all respects except the suicidal thoughts and was designed to assess how this symptom affected the public's response. The chronic schizophrenia vignette was designed to assess the response to someone with a severe long-standing disorder, where acceptance seemed less likely. Respondents were also randomly assigned to receive either male ("John") or female ("Mary") versions of the vignette. The vignettes have been given in an earlier publication [13]. After being presented with the vignette, respondents were questioned about what was wrong with the person, how they could be helped, the likely helpfulness of a range of interventions, the likelihood of recovery, knowledge of causes and risk factors, beliefs associated with stigma and discrimination, contact with people like those in the vignette, and the health of the respondent.

The only questions of relevance here are those concerned with causes and risk factors [1]. These questions were: "There are many people in the community who suffer from problems like John's. The next few questions are about possible causes of this sort of problem developing in anybody. How likely do you think each of the following is to be a reason for such problems? Could a virus or other infection, be a reason for these sorts of problems? How likely is an allergy or reaction to be the cause? Day-to-day problems such as stress, family arguments, difficulties at work or financial difficulties? The recent death of a close friend or relative? Some recent traumatic event such as bushfires threatening your home, a severe traffic accident or being mugged? Problems from childhood such as being badly treated or abused, losing one or both parents when young or coming from a broken home? How likely is it that these sorts of problems are inherited or genetic? Is being a nervous person likely to be a reason? Having weakness of character?" Response options to these questions were: very likely, likely, not likely, depends, don't know.

Then followed questions about risk factors: "The next few questions seek your opinion about whether there are some people in the community who are more likely to have these problems and others who are perhaps less likely. Do you think that women would be more likely or less likely than men to suffer these sorts of problems? Would young people, under 25 years of age, be more likely or less likely? Would older people, those aged over 65, be more likely or less likely? Would poor people be more likely or less likely to suffer these sorts of problems? Unemployed people? Divorced or separated people? Would single people, who have never been married or in a long-term relationship be more likely or less likely?". The response options were: More likely, less likely, no difference, depends, don't know.

### The Japanese survey

A survey manual supplied from Australia was translated into Japanese and entrusted to Yamate Information Processing Center Ltd. for use with the target population aged 20–69 years, as a rule using the same procedures as Australia. The survey questionnaire, which was developed by the Australian researchers (AFJ, HC, KMG), was tentatively translated into Japanese. Then a native English translator, who had not seen the original English text, translated the Japanese version back into English. By comparing the two English versions, it was possible to confirm the accuracy of the original translation. There were no significant differences between the original text and the reverse translation. Finally, a Japanese version of the questionnaire was produced, which involved formatting the text into Japanese style and making slight wording adjustments. The names of the characters in the vignettes were translated into the Japanese style, viz. "A-o"(putting an o sound at the end is often used for a man's name) or "B-ko" (putting ko at the end is often used for a woman's name), instead of "John" or "Mary" which were used in the English text.

As well as the questions taken from the Australian survey, the Japanese survey asked questions concerning such issues as psychiatric health and welfare policy, the bodies implementing related services, the existence of action groups, and the change in the Japanese name for schizophrenia by the Japanese Society of Psychiatry and Neurology. These additions were made to clarify the current Japanese situation and issues in related fields. Further, an original Japanese manual was also created and adopted for use concerning points of interest in the implementation of home visits.

The survey method used was home visit interviews. It was not feasible to do a national survey of randomly selected households in Japan because of constraints of human resources, funding and time. It was therefore decided to sample a range of areas that differed in whether they were large or small cities, whether the area had many psychiatric patients or not, and whether the area had a high suicide rate or not. Using this approach, Japan was divided into 5 areas and 5 research sites were selected in each of these areas, giving a total of 25 geographic sites. As the survey was conducted during the winter, and because it was difficult to ensure that there would be enough survey interviewers, implementation in Hokkaido and Shikoku prefectures proved troublesome. Additional reasons for selection of the 25 regional sites were that they were places of comparatively high population within the relevant regions, the survey interviewers could use public transport, and the urban areas involved no particular inconveniences for the researchers to visit within a certain range using public transportation. 80 households were selected from each site, giving a total of 2000. At each site there were 4 interviewers who took responsibility for 20 households each. The survey was conducted over the period from 19 November to 12 December 2003. Each of the four vignettes was received by 250 people. Half received a male version of a vignette and half the female version.

At the start of the survey, an explanatory meeting was held for the survey interviewers in each region. As many members of the research team as possible attended these explanatory meetings. Eighty-five survey interviewers were recruited for this research with an average age of 50 and an average of 17 years' experience of survey interviewing in various types of surveys. The areas for the survey interviewers to canvass were allocated on the basis of where they lived. The question of where the individual survey interviewers should go was determined mutually among the survey interviewers themselves, and by the head survey interviewer (supervisor). As a rule, one survey interviewer conducted 20 interviews, but this was considerably flexible, given the number of years of individual experience and what the individual survey interviewer could handle. The interviews were conducted according to the following procedure: visit the target's home and present the written greetings and request (a draft had been prepared by certain survey bodies, which was put into final form after checks by the research team members), then explain the details of the survey using the documents, ask the target for their participation in the research, start the interview and follow through to completion, check that nothing had been omitted from the survey responses, and hand over the remuneration (1000 yen cash voucher). Data were not collected on the refusal rate for this survey because the emphasis was on achieving the quotas of respondents to fit the required age and gender distribution.

### The Australian survey

A household survey was carried out on Australian adults aged 18 years or over by the company AC Nielsen. Households were sampled from 250 census districts covering all states and territories and metropolitan and rural areas. Up to 5 call backs were made to metropolitan selections and 3 to non-metropolitan selections. Interviewers attempted to interview the person in each household with the most recent birthday. To achieve a target sample of 4,000 interviews with adults aged 18 years or over, visits were made to 28,947 households. The outcome of these visits was: no contact after repeated visits 14,630; vacant house or lot 306; refused 7,815; person sampled within household temporarily unavailable 1,132; no suitable respondent in household 287; did not speak English 383; incapable of responding 213; and unavailable for the duration of the survey 181. The achieved sample was 3998 persons, with 1001 receiving the depression vignette, 999 the depression with suicidal thoughts vignette, 997 the early schizophrenia vignette, and 1001 the chronic schizophrenia vignette. The interviews were carried out between November 2003 and February 2004.

In addition to the common core component, the Australian survey interview had questions about awareness of depression in the media and about Australia's national depression initiative.

Ethics approval was given by the Human Research Ethics Committee of the Australian National University.

### Statistical analysis

Data were pooled across male and female versions of each vignette and percent frequencies calculated. For the Japanese survey, percentage frequencies and 95% CIs were calculated using unweighted data with SPSS 12.0. For the Australian survey, percentages were calculated applying survey weights to give better population estimates. Ninety-five percent CIs were estimated using the Complex Samples procedure in SPSS 12.0. This procedure takes account of sampling weights and geographic clustering in the sample.

Because of the very different cultures of Japan and Australia, it is possible that any differences in question endorsement rates might be due to subtleties of language or to the social rules applying to the interview situation, as well as to genuine differences in beliefs about treatment and outcome. For this reason, we have not relied on statistical significance of percentage differences between countries, but rather on the broad patterns of responses, particularly where percent endorsement was ordered very differently across questions.

## Results

### Characteristics of the samples

Table 1 shows the age and gender distributions of the Japanese and Australian samples. Comparing the Japanese sample to the national population in the same age groups (2003 data), there was an under-representation of 50–59 year olds (20% vs 22.4%) and an over-representation of 60–69 year old males (10% vs 8.9%). Other age-gender groups showed less than 1% discrepancy.

**Table 1:** Age and gender distribution of the Japanese and Australian samples

**Age group**	**Japanese males %**	**Japanese females %**	**Japanese total %**	**Australian males %**	**Australian females %**	**Australian total %**
18–19	-	-	-	1.5	1.6	3.0
20–29	10.0	10.0	20.0	5.6	8.1	13.8
30–39	10.0	10.0	20.0	7.7	11.8	19.4
40–49	10.0	10.0	20.0	8.6	11.0	19.7
50–59	10.0	10.0	20.0	6.9	9.5	16.3
60–69	10.0	10.0	20.0	4.8	8.0	12.7
70+	-	-	-	6.2	8.9	15.1
Total	50.0	50.0	100.0	41.2	58.8	100.0

Comparing the Australian sample to the national population, there was an under-representation of males and of younger adults, but the sample was close to the population in marital status, country of birth and education. For the Australian sample, weights were used to correct for these biases.

### Beliefs about causes and risk factors

Table 2 shows the results on beliefs about causes. In this table, the percentages pertain to each question asked separately, so that respondents could endorse any number of factors as likely causes. In both countries there was a common belief in social causes, such as day-to-day problems, death of someone close, traumatic event, and problems from childhood. This belief was common across all vignettes. The major differences were that the Australians were more likely to believe in virus or infection, allergy, and inherited or genetic, while the Japanese were more likely to endorse nervous person and weakness of character.

**Table 2:** Percentage (and 95% CI) of Japanese and Australian respondents giving each cause as "very likely" or "likely" for the person described in the vignette

**Cause**	**Depression Vignette**	**Depression/Suicidal Vignette**	**Early Schizophrenia Vignette**	**Chronic Schizophrenia Vignette**
**Virus or infection**				
Japanese	6.2 (4.1–8.3)	6.6 (4.4–8.8)	7.2 (4.9–9.5)	7.2 (4.9–9.5)
Australian	50.5 (47.1–54.0)	41.4 (38.2–44.7)	32.1 (29.0–35.4)	33.6 (30.7–36.6)
**Allergy**				
Japanese	10.2 (7.5–12.9)	11.4 (8.6–14.2)	12.6 (9.7–15.5)	9.4 (6.8–12.0)
Australian	44.9 (41.4–48.5)	37.6 (34.3–41.0)	31.5 (28.4–34.7)	28.4 (25.5–31.5)
**Day-to-day problems**				
Japanese	93.6 (91.4–95.8)	91.8 (89.4–94.2)	92.0 (89.6–94.4)	91.2 (88.7–93.7)
Australian	96.8 (95.2–97.9)	95.7 (94.2–96.9)	89.6 (87.6–91.2)	86.6 (84.3–88.7)
**Death of someone close**				
Japanese	79.8 (76.3–83.3)	81.4 (78.0–84.8)	73.4 (69.5–77.3)	74.0 (70.1–77.9)
Australian	96.3 (94.6–97.5)	94.8 (93.1–96.0)	87.4 (85.2–89.4)	83.3 (80.7–85.6)
**Traumatic event**				
Japanese	82.6 (79.3–85.9)	79.6 (76.1–83.1)	78.2 (74.6–81.8)	80.8 (77.3–84.3)
Australian	93.9 (91.8–95.4)	92.7 (90.7–94.2)	86.5 (84.1–88.6)	82.8 (80.2–85.1)
**Problems from childhood**				
Japanese	81.0 (77.5–84.5)	82.0 (78.6–85.4)	88.2 (85.4–91.0)	89.0 (86.2–91.8)
Australian	91.3 (89.2–93.1)	95.0 (93.2–96.3)	90.8 (88.8–92.5)	91.4 (89.4–93.0)
**Inherited or genetic**				
Japanese	34.6 (30.4–38.8)	34.0 (29.8–38.2)	34.2 (30.0–38.4)	43.8 (39.4–48.2)
Australian	68.0 (64.8–71.0)	68.4 (65.3–71.3)	70.0 (66.8–73.0)	73.7 (70.6–76.6)
**Nervous person**				
Japanese	81.4 (78.0–84.8)	77.4 (73.7–81.1)	74.0 (70.1–77.9)	81.8 (78.4–85.2)
Australian	67.9 (64.6–70.9)	65.6 (62.3–68.7)	58.1 (54.4–61.7)	56.9 (53.6–60.2)
**Weakness of character**				
Japanese	73.6 (69.7–77.5)	69.2 (65.1–73.3)	73.4 (69.5–77.3)	82.0 (78.6–85.4)
Australian	43.0 (39.7–46.3)	46.1 (42.8–49.3)	39.7 (36.5–42.9)	35.1 (31.6–38.8)

Tables 3 and 4 show the data on beliefs about risk factors. Table 3 gives the percentages believing a group is more at risk and Table 4 the percentages believing a group is less at risk. As in Table 2, each group was asked about separately, so that respondents could endorse any number as more likely or less likely to be at risk. In both countries, the risk factors most strongly believed in across vignettes were being unemployed and divorced/separated, although these beliefs were more common in Australia. There were a number of cross-national differences. The Australians tended to believe that the young and the poor were more at risk of depression, but these were not seen as higher risk groups by the Japanese. In fact, the Japanese public tended to see the poor as having lower risk of depression. For schizophrenia, the Australians saw the young as having higher risk for early schizophrenia, and the poor as having higher risk for both early and chronic schizophrenia. Likewise the Japanese saw the young as having higher risk for early schizophrenia, but they again tended to see the poor as having lower risk.

**Table 3:** Percentage (and 95% CI) of Japanese and Australian respondents rating each group in the population as "more likely" to experience the problem described in the vignette

**Group More Likely at Risk**	**Depression Vignette**	**Depression/Suicidal Vignette**	**Early Schizophrenia Vignette**	**Chronic Schizophrenia Vignette**
**Women**				
Japanese	29.4 (25.4–33.4)	24.2 (20.4–28.0)	21.4 (17.8–25.0)	23.0 (19.3–26.7)
Australian	26.8 (23.9–30.0)	27.2 (24.4–30.2)	21.1 (18.5–23.9)	14.7 (12.6–17.1)
**Young**				
Japanese	24.2 (20.4–28.0)	24.4 (20.6–28.2)	40.4 (36.1–44.7)	26.2 (22.3–30.1)
Australian	42.5 (39.1–46.0)	48.2 (45.0–51.4)	55.3 (52.0–58.6)	28.4 (25.6–31.4)
**Old**				
Japanese	23.4 (19.7–27.1)	21.2 (17.6–24.8)	15.0 (11.9–18.1)	29.2 (25.2–33.2)
Australian	28.6 (25.8–31.5)	29.1 (26.3–32.1)	22.0 (19.4–24.9)	38.3 (35.0–41.7)
**Poor**				
Japanese	14.8 (11.7–17.9)	13.2 (10.2–16.2)	7.4 (5.1–9.7)	19.6 (16.1–23.1)
Australian	52.6 (49.2–56.0)	52.1 (48.6–55.5)	38.9 (35.7–42.3)	37.8 (34.4–41.2)
**Unemployed**				
Japanese	58.4 (54.1–62.7)	50.8 (46.4–55.2)	41.0 (36.7–45.3)	50.6 (46.2–55.0)
Australian	76.3 (73.0–79.3)	76.7 (73.6–79.4)	62.7 (59.4–65.9)	54.9 (51.4–58.3)
**Divorced/separated**				
Japanese	48.6 (44.2–53.0)	43.6 (39.2–48.0)	37.2 (32.9–41.5)	39.6 (35.3–43.9)
Australian	69.6 (66.2–72.8)	64.3 (60.9–67.6)	53.6 (50.3–57.0)	44.3 (40.9–47.8)
**Single**				
Japanese	18.2 (14.8–21.6)	22.8 (19.1–26.5)	22.8 (19.1–26.5)	24.6 (20.8–28.4)
Australian	23.9 (21.0–27.1)	27.1 (24.2–30.1)	22.0 (19.2–25.0)	25.1 (22.2–28.2)

**Table 4:** Percentage (and 95% CI) of Japanese and Australian respondents rating each group in the population as "less likely" to experience the problem described in the vignette

**Group Less Likely at Risk**	**Depression Vignette**	**Depression/Suicidal Vignette**	**Early Schizophrenia Vignette**	**Chronic Schizophrenia Vignette**
**Women**				
Japanese	21.6 (18.0–25.2)	22.8 (19.1–26.5)	24.0 (20.2–27.8)	24.8 (21.0–28.6)
Australian	12.0 (9.7–14.7)	13.6 (11.5–16.0)	12.8 (10.8–15.1)	18.7 (16.4–21.3)
**Young**				
Japanese	24.0 (20.2–27.8)	20.8 (17.2–24.4)	14.6 (11.5–17.7)	27.6 (23.7–31.5)
Australian	19.5 (16.8–22.4)	18.8 (16.4–21.4)	10.9 (8.9–13.2)	30.6 (27.5–33.9)
**Old**				
Japanese	30.2 (26.2–34.2)	25.0 (21.2–28.8)	38.6 (34.3–42.9)	27.4 (23.5–31.3)
Australian	34.8 (31.5–38.2)	37.2 (34.2–40.3)	43.2 (39.8–46.7)	25.2 (22.5–28.1)
**Poor**				
Japanese	23.4 (19.7–27.1)	19.6 (16.1–23.1)	26.4 (22.5–30.3)	23.4 (19.7–27.1)
Australian	8.5 (6.6–10.8)	7.4 (6.0–9.2)	9.1 (7.3–11.3)	7.2 (5.7–9.1)
**Unemployed**				
Japanese	13.0 (10.0–16.0)	11.0 (8.2–13.8)	14.8 (11.7–17.9)	15.6 (12.4–18.8)
Australian	4.4 (3.0–6.2)	4.6 (3.5–6.2)	5.4 (4.1–7.1)	5.5 (4.1–7.5)
**Divorced/separated**				
Japanese	12.2 (9.3–15.1)	13.6 (10.6–16.6)	14.0 (10.9–17.1)	16.0 (12.8–19.2)
Australian	4.1 (3.0–5.7)	5.5 (4.2–7.3)	5.2 (3.9–6.9)	6.7 (5.1–8.7)
**Single**				
Japanese	19.2 (15.7–22.7)	13.0 (10.0–16.0)	13.6 (10.6–16.6)	20.4 (16.9–23.9)
Australian	21.4 (18.8–24.4)	19.2 (16.6–22.2)	17.7 (15.2–20.4)	17.1 (14.9–19.6)

## Discussion

The present findings from Japan and Australia support earlier work from several countries showing a predominant belief in social causes. These causes include day-to-day problems, death of someone close, traumatic events, and problems from childhood. The findings on risk factors are generally consistent with those on causes, with unemployment and divorce/separation widely seen to be risk factors in both countries. While a belief in social causes and risk factors was found in both countries, it was generally more common in Australia than in Japan. As far as depression is concerned, this belief is realistic because there is substantial evidence supporting social factors in the causation of depression [14]. However, for schizophrenia, social factors are of less importance, having an influence only on those who are genetically vulnerable [15].

The findings on poverty as a risk factor were an exception to the general trend of beliefs in social factors. While the Australians tended to see the poor as having higher risk, particularly for depression, for the Japanese the trend was in the opposite direction, with more people believing that the poor would have lower risk. The reason for this difference is unknown. It may reflect inaccurate beliefs in one country or else a true difference in risk factors between countries. In Australia, poverty is known to be associated with both depression and psychotic disorders [16,17], although there is debate about the causal pathways. However, in one Japanese study of depression in the workplace, poor economic status was not associated with increased risk [18]. Furthermore, a Japanese incidence study of schizophrenia found that features of residential areas that are associated with higher incidence in Western countries do not necessarily have the same association in Japan [19].

As well as a frequent public belief about social causes in both countries, there was also a common belief in personal vulnerability factors. However, the endorsement of personal vulnerability factors tended to take a different form in each country. The Japanese were more likely to believe in the role of the trait characteristics of nervous person and weakness of character, while Australians were more likely to endorse the role of genetics. Being a nervous person could be regarded as a lay description of the personality trait of neuroticism, which is a major risk factor for depression. There is also some evidence that neuroticism is a risk factor for schizophrenia [20]. However, the belief in weakness of character is of more concern, because this is a more stigmatizing explanation which could make people less likely to disclose that they are experiencing a mental disorder and to seek professional help. It has been noted that "Japanese patients are reluctant to openly discuss disturbances of mood, since these are considered to be indicative of personal weakness rather than treatable medical conditions" [21]. For Japanese people, the implication is that the disorder is the person's fault. In Australia, the belief in weakness of character as a cause has declined since the earlier survey in 1995, particularly for schizophrenia [22]. This change may have been affected by efforts to reduce the stigma of mental disorders.

The strong endorsement of genetics by Australians represents a major change from 8 years earlier, from around half the population in 1995 to around two-thirds in 2003–04 [22]. A possible reason for this change is the publicity surrounding the human genome project and the role of genes in health generally. Why this belief is weaker in Japan is unclear. However, in both countries genetics was seen to be more important for the chronic schizophrenia vignette than for the other vignettes, suggesting a greater genetic attribution for severe or chronic mental disorders.

Another difference between the two countries was that Australians were more likely to believe in the role of virus or infection and allergy. It is not clear why these beliefs are more prominent in Australia. However, such beliefs appear to be stable over time, because the percentages endorsing these causes are very similar to an Australian national survey carried out 8 years earlier [22]. These beliefs were most common for the vignette of depression without suicidal thoughts, which is the least severe of the cases presented. It may be that they reflect interpretations of the vignette as being a physical disorder or a secondary reaction to a physical disorder.

Taking all the findings together, there is some broad similarity between public and professional beliefs about causation, in that mental disorders are seen to be influenced by a combination of personal vulnerability and environmental triggers. The major difference would appear to be the greater use of morally judgmental attributions of personal vulnerability by the public compared to the more objective attributions of professionals. In this regard, the Australian public's views appear closer to those of professionals than do the Japanese public's.

### Limitations

We have previously discussed some of the limitations of this work [13]. These relate to the methodology of the surveys, in particular the non-contact and refusal rate in the Australian survey and the lack of truly national coverage of the Japanese one. Furthermore, both surveys lack data on the characteristics of refusers. We also recognise the problems of making cross-national comparisons between two very different cultures. There will inevitably be subtleties of meaning and cultural factors operating within a structured household survey which could affect the results in unknown ways. Finally, the survey used closed rather than open-ended questions, which may have suggested responses that the participants would not have thought of spontaneously.

## Conclusion

In both Japan and Australia, the public have a predominant belief in social causes and risk factors for mental disorders. However, there are also some major differences between the countries, with Australians having a stronger belief in infections, allergies and genetics, while Japanese have a stronger belief in being a nervous person and weakness of character. The latter belief is of particular interest because is may be associated with greater stigma and reduce the likelihood of seeking professional help and support from others. Reducing the belief in weakness of character as a cause would be a suitable target for mental health literacy campaigns. This is probably easier to achieve for depression, where there are both contemporary and historical figures who have suffered from depression, yet are perceived as being strong in character.

## Competing interests

The author(s) declare that they have no competing interests.

## Authors' contributions

YN provided overall supervision of the research and provided comments on the manuscript.

AFJ co-designed the Australian survey, analyzed the Australian data, and co-wrote the manuscript.

YK provided specific guidance on the Japanese survey, including participation in survey interviewer training, and co-wrote the manuscript.

HC co-designed the Australian survey and provided comments on the manuscript.

HN provided specific guidance on the Japanese survey, including participation in survey interviewer training, and co-wrote the manuscript.

KMG co-designed the Australian survey and provided comments on the manuscript.

## Pre-publication history

The pre-publication history for this paper can be accessed here:


